# Hypoxia and Oxidative Stress Are Associated with Reduced Fetal Growth in Twin and Undernourished Sheep Pregnancies

**DOI:** 10.3390/ani8110217

**Published:** 2018-11-19

**Authors:** Francisco Sales, Oscar A. Peralta, Eileen Narbona, Sue McCoard, Mónica De los Reyes, Antonio González-Bulnes, Víctor H. Parraguez

**Affiliations:** 1 INIA-Kampenaike, Punta Arenas 6212707, Chile; fsales@inia.cl; 2 Faculty of Veterinary Sciences, University of Chile, Santiago 8820808, Chile; operalta@uchile.cl (O.A.P.); eileen.narbona@gmail.com (E.N.); mdlreyes@uchile.cl (M.D.l.R.); 3 AgResearch Grasslands, Palmerston North 4442, New Zealand; Sue.McCoard@agresearch.co.nz; 4 INIA-Madrid, Ciudad Universitaria s/n, 28040 Madrid, Spain; agbulnes@gmail.com; 5 Facultad de Veterinaria, Universidad Complutense de Madrid, Ciudad Universitaria s/n, 28040 Madrid, Spain; 6 Faculty of Agricultural Sciences, University of Chile, Santiago 8820808, Chile

**Keywords:** ovine gestation, fetal growth, nutrition, oxygen supply, placental–fetal redox

## Abstract

**Simple Summary:**

Twin gestations in sheep are economically more important than single ones; however, twins are born with lower weight, resulting in higher mortality and lower postnatal growth. Chilean Patagonia is very important for sheep production, but its harsh environment results in low food availability and cold and windy conditions during gestation and lambing periods, with a consequent great mortality of twins (~40%). We postulate that the restriction of fetal growth in twin and undernourished sheep pregnancies is associated with fetal hypoxia and oxidative stress. To prove this, single- and twin-bearing ewes were maintained under Patagonian field conditions and offered only natural pasture (undernourished) or natural pasture plus concentrate supplementation (well nourished). Near term, blood gases and oxidative status were evaluated in cord blood, and fetal biometric traits and placental weight were obtained after cesarean section. Both maternal undernutrition and twinning led to decreased oxygen supply to the fetuses, which was associated with decreased intrauterine growth. Moreover, twinning increased oxidative stress at the feto-placental unit, which might also contribute to the restriction of fetal growth. These results highlight the importance of maternal nutrition, especially for those ewes bearing multiples, and opens new possibilities for nutritional or antioxidant interventions for preventing fetal hypoxia and oxidative stress.

**Abstract:**

Low birth weight has profound implications for perinatal mortality and morbidity in lambs, causing higher mortality and lower growth potential. Low birth weight, as a consequence of fetal growth restriction, occurs in undernourished and multiple pregnancies, where hypoxia and oxidative stress could play a critical role. Our aim was to establish the effects of nutritional deprivation and pregnancy rank on fetal growth, oxygenation, and oxidative status in sheep pregnancies under extensive Patagonian conditions. At 30 days after mating, single- and twin-bearing ewes were offered only natural pasture (undernutrition group) or natural pasture plus concentrate supplementation (well-nourished group). At day 140 of gestation, blood gases and redox status were evaluated in venous cord blood, and fetal biometric characteristics were obtained after cesarean section. Both maternal undernutrition and twinning led to decreased oxygen supply to the fetuses (*p* = 0.016 and *p* = 0.050, respectively), which was associated with decreased intrauterine growth (r = 0.446, *p* < 0.01). Moreover, twinning increased oxidative stress in cord blood (*p* < 0.05), which might also contribute to fetal growth restriction. These results reinforce the importance of maternal nutrition, especially for those ewes bearing multiples, and opens new possibilities for nutritional or antioxidant interventions for preventing fetal hypoxia and oxidative stress.

## 1. Introduction

The Chilean sheep flock consists of around 4 million head of breeding ewes, with more than 56% of them located in the Patagonian region [1]. Extensive grazing systems with natural pastures are the sole feed source. This region experiences climatic extremes, particularly, high winds, cold temperatures, and low rainfall. As a result, pastures often have low availability of and low-quality forage with both low protein and low energy content, particularly during winter, when the ewes are pregnant [2]. Thus, although the stocking rate is low (0.6–0.9 sheep per hectare), breeding ewes often experience a nutritional restriction of about 30% of their requirements [3], which may lead to fetal growth restriction, resulting in low birth weight. Furthermore, around 15% of the pregnancies are twins [4], characterized by perinatal mortality in the Patagonian environment of up to 40%, with most mortality occurring in the first 72 h of postnatal life [5]. Therefore, new nutritional and management strategies are required to improve lamb survival, especially in twin pregnancies, and address this important welfare and economic issue.

Maternal undernutrition negatively impacts fetal growth, especially in twin pregnancies [6]. Furthermore, in twin pregnancies, total placental mass, number of placentomes per fetus, and, thus, capacity for nutrient transfer are reduced [7], therefore, fetal growth restriction (FGR) in twin pregnancies is also frequent [8,9,10]. Fetal growth restriction as a result of maternal undernutrition [11] or twinning [7,12] has profound implications for fetal and postnatal health and wellbeing, according to what is known as fetal or developmental programming [13]. Reproduction and production are also affected, because low-birth-weight individuals often have delayed sexual development, lower fertility [14], higher mortality, lower growth and feed efficiency, poorer carcass conformation, and lower meat yield and quality than their normal-weight mates, resulting in economic losses [15,16,17,18].

Evidence suggests that the pathogenesis of fetal growth restriction seems to be related not only to inadequate nutrient supply, but also to reduced oxygen delivery to the fetus and, therefore, hypoxia [19,20,21]. Furthermore, hypoxia induces oxidative stress and aggravates the effects of FGR, as found in hypoxic pregnancies [22]. The aim of this study was to establish the effects of naturally occurring maternal undernutrition and pregnancy rank on fetal growth, oxygenation status, and oxidative stress biomarkers in sheep pregnancies under Patagonian extensive production conditions.

## 2. Materials and Methods

### 2.1. Ethics Statement

Animal handling was done in accordance with the Guide for Care and Use of Laboratory Animals (Eighth Edition, National Research Council, National Institute of Health, USA). The experimental procedure was approved by the Bioethics Review Committee of the Faculty of Veterinary Sciences of the University of Chile (Protocol # 11-2016) and the Bioethics Committee of the Instituto Nacional de Investigaciones Agropecuarias (INIA, Ministry of Agriculture), as institutions where the work was carried out, and the Bioethics Advisory Committee of the Chilean National Commission for Scientific and Technological Research (CONICYT, Chile), as the funder of the project.

### 2.2. Animals and Experimental Procedure

The present field study was carried out at the INIA research farm, 65 km north from Punta Arenas, in the Magellan Region, Chilean Patagonia (Lat 52°36′; Lon 70°56′). The trial involved 32 Corriedale ewes, 4–6 years old, with body weight of 62.8 ± 3.5 kg and body condition score (BCS, scale 0–5) of 2.0–2.5 [23] at mating, carrying a singleton (n = 16) or twins (n = 16). These females were selected from the INIA experimental flock, whose estrous cycles were synchronized with intravaginal CIDR devices (CIDR G^®^, Pfizer, Santiago, Chile) maintained for 12 days and a single i.m. dose of 300 IU eCG (Novormon^®^, Syntex, Buenos Aires, Argentine) at CIDR removal to increase the ovulation rate and, therefore, twinning for the study. Mating was carried out in a group of 200 ewes, using 20 fertile Suffolk rams with chests painted with a solution containing food-grade oil with colored earth. The exact day of service of the ewes was identified by daily visual inspection of the colored rumps of the ewes mated on the same day and, therefore, the experimental group was chosen from ewes mated during about two days.

The study was carried out under standard extensive rangeland conditions. The experimental model had a 2 × 2 factorial design with two feeding regimes (grazing and grazing plus supplementation) and two pregnancy ranks (single and twin). The selection of the animals by rank was achieved by ultrasound examination at Day 30 of pregnancy (~20% of the total length of ovine pregnancy, estimated in a mean of 148 days). These sheep were randomly divided in two equal groups in each pregnancy rank and tagged with two different colors, according to each nutritional treatment (fed only prairie or prairie plus concentrate supplementation), for easier identification. The animals were maintained during gestation on natural pasture (*Festuca gracillima–Chiliotrichium diffusum*; CP: 3.3%, ME: 1.9 Mcal/kg, TDN: 45%); the stocking rate was 0.9 sheep per hectare, and the availability of dry matter was 525 kg per hectare, representing Patagonian prairie conditions. Additionally, the group of supplemented ewes received a concentrate ration daily (CP: 17.0%; ME: 3.0 Mcal/kg), aiming to ensure a total dam live weight increase in pregnancy at a level similar to that of the expected conceptus mass [24]. Every month, body weight and BCS were assessed in all the animals in order to adjust the supplementation level. Hence, four experimental groups were generated according to pregnancy rank and plane of nutrition: singletons (S) offered only natural pasture (SP, n = 8 ewes), twins (T) offered only natural pasture (TP, n = 8 ewes), SP offered concentrated supplementation (SP+S, n = 8 ewes), and TP offered concentrated supplementation (TP+S, n = 8 ewes).

Sampling of the feto-placental unit was performed near term (140 ± 0.7 days of gestation; ~95% of the total length of ovine pregnancy). A cesarean section was performed under spinal anesthesia by administration of 2 mL of 2% lidocaine hydrochloride (Lidocalm^®^, Drag Pharma, Santiago, Chile). Incision of the pregnant uterine horns allowed blood sampling with heparinized syringes (1000 IU mL^−1^ solution), via the umbilical vein of each fetus. Simultaneously, a maternal carotid arterial blood sample was drawn. One sample of fetal blood (1 mL) and the maternal sample were used for direct and immediate evaluation of the oxygenation status; a second fetal sample (5 mL) was centrifuged at 1200× *g* for 5 min at 4 °C, and plasma was harvested and stored in liquid nitrogen until assayed for oxidative stress biomarkers. The fetuses and ewe were immediately euthanized by barbiturate overdose (Opet^®^, Pro-Vet, Santiago, Chile), and the fetuses and placentas were extracted. Fetal body weight (BW), crown–rump length (CRL), thorax perimeter, fore- and hind-leg length, and total placental weight for each individual fetus were obtained. Furthermore, fetal brain and liver were dissected and weighed for the evaluation of the brain/liver ratio. Fetal body mass index (BMI, BW/CRL^2^, kg/m^2^) and G index (BW/CRL^1.5^, kg/m^1.5^) were estimated according to Gootwine [25].

### 2.3. Assessment of Maternal and Feto-Placental Oxygenation Status

Partial pressure of oxygen (PO_2_), partial pressure of carbon dioxide (PCO_2_), hemoglobin (Hb) concentration, Hb saturation by oxygen (SatHb) and pH value were measured by using an IL Synthesis 25™ gas analyzer (Instrumentation Laboratory, Lexington, MA, USA), adjusted to ovine body temperature [26]. For samples from the umbilical vein, the oxygen content (O_2_ cont.) was also calculated, in accordance to Danielson et al. [27].

### 2.4. Assessment of Oxidative Stress Biomarkers

Plasma malondialdehyde concentrations and total antioxidant capacity were used for the evaluation of the feto–placental redox status. The quantification of malondialdehyde was performed using a colorimetric kit (TBARS, TCA method, Assay Kit, Cayman Chemical Company, Ann Arbor, MI, USA), according to the manufacturer’s instructions. The absorbance was read at 540 nm with a microplate reader (Perlong DNM-9602, Nanjing Perlove Medical Equipment Co. Ltd., Nanjing, China). The assessment of total antioxidant capacity in plasma was performed using a colorimetric Antioxidant Assay Kit (Cayman Chemical Company, Ann Arbor, MI, USA), according to the instructions of the manufacturer. The absorbance was read at 405 nm with the microplate reader. Both assay kits have been previously used with ovine plasma [28].

### 2.5. Statistical Analysis

Comparisons among groups were done by analysis of variance, using the general linear model procedure of SAS (GLM; SAS Institute Inc., Cary, NC, USA), after normality testing of the data. Maternal body weight and body condition score changes were analyzed through repeated measures analysis, considering pregnancy rank (single or twin) and nutrition (natural pasture or natural pasture plus supplement) as independent effects. Fetal data were analyzed using a linear model including the fixed effects of pregnancy rank, the nutritional plane, the sex of the fetus, as well as their interactions and the random effects of ewes, to adjust for twin pregnancy. The effects of fetal sex and of the interactions of treatment and sex were not significant (*p* > 0.05) and, therefore, were removed from the model. In addition, Pearson correlations between cord blood PO_2_, O_2_ content, and oxidative stress biomarkers with fetal and placental traits were also calculated. The differences were considered significant when *p* < 0.05. The results were expressed as means ±SEM.

## 3. Results

### 3.1. Effects of Nutrition and Twinning on Maternal Weight and Body Condition

**Figure 1:**
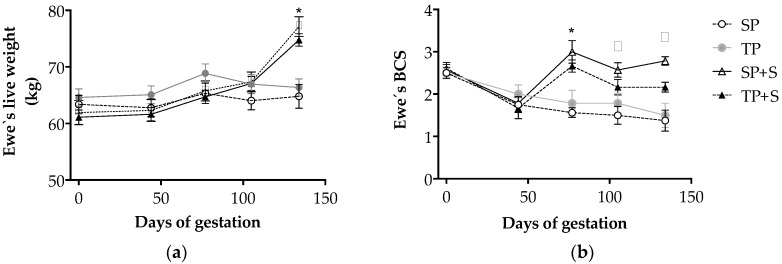
Live weight (**a**) and body condition score (BCS) (**b**) during fetal growth restriction pregnancy in ewes bearing single and twin fetuses, maintained under adequate or deprived nutritional planes. Groups are as follows: SP, pregnant ewes bearing a single fetus, consuming only natural pasture; TP, pregnant ewes bearing twin fetuses, consuming only natural pasture; SP+S, SP ewes consuming natural pasture plus concentrate supplementation; TP+S, TP ewes consuming natural pasture plus concentrate supplementation. Asterisks indicate a significant difference between the supplemented and the non-supplemented groups at the same sampling point; ƒ indicates a significant difference between the supplemented and the non-supplemented groups at the same sampling point, when TP and TP+S are similar; δ indicate a significant difference between the supplemented and the non-supplemented groups at the same sampling point with SP+S also different from TP+S (ANOVA, *p* < 0.05). A time when the effects of pregnancy rank and nutritional status interaction were evident (*p* < 0.001) was identified for both traits.

On the basis of pregnancy rank and nutrition plane interaction, a divergence in ewe live weight was observed at 100–140 days of pregnancy (*p* < 0.001; Figure 1a), resulting in a 14% difference between treatment groups by Day 140 (*p* < 0.05). During the trial, supplemented ewes exhibited positive weight gain (14.5 kg), whereby unsupplemented ewes exhibited only a small increase in live weight (1.6 kg), independent of the pregnancy rank (*p* > 0.05). The supplemented ewes consumed, on average, 58 kg of concentrate.

A time affected by pregnancy rank and nutrition plane interaction was observed for BCS (*p* < 0.001; Figure 1b), with a divergence evident by Day 75 of pregnancy, and, thereafter, when a greater BCS was evident in supplemented compared to unsupplemented ewes (*p* < 0.05). Furthermore, while pregnancy rank had no effect on BCS in unsupplemented ewes, SP+S ewes had a greater BCS at Day 100 and Day 140 of pregnancy compared to TP+S ewes (*p* < 0.05).

### 3.2. Effects of Nutrition and Twinning on Fetal and Maternal Traits

A total of 48 fetuses, 25 females and 23 males, were studied. Although the females were numerically lighter than the males (3.83 ± 0.12 versus 4.01 ± 0.17 kg), no significant differences between the sexes were evident (*p* > 0.05). Therefore, the following results consider females and males together. Fetal body weight was affected by both nutritional plane and pregnancy rank, while no interaction was observed (Table 1). Fetuses from unsupplemented ewes were lighter than those from supplemented ewes (3.74 ± 0.14 versus 4.11 ± 0.13; *p* < 0.05), and twins were lighter than singletons (3.66 ± 0.11 versus 4.39 ± 0.15; *p* < 0.001). Undernutrition resulted in only a small decrease in the fetal body weight of singleton fetuses (~5%), whilst twinning was the main factor for the appearance of fetal growth restriction, whereby 12.5% and 44.4% of the fetuses in the SP and TP groups, respectively, exhibited body weight below the 10th percentile for their gestational age.

The assessment of fetal morphometry also showed effects of both nutritional plane and twinning, but no nutritional plane by pregnancy rank interaction was observed. Crown–rump length, thorax perimeter, and forelimb lengths were greater in singletons compared to twin fetuses and in fetuses from supplemented versus unsupplemented ewes (*p* < 0.05; Table 1). Fetal hind limb length was greater in singletons compared to twin fetuses (*p* < 0.01) but was unaffected by maternal supplementation (*p* > 0.05). Fetal brain weight was not affected by rank or nutritional status, nor by their interaction (*p* > 0.05). Fetal liver weight was higher in singletons compared to twins, while fetuses from supplemented dams showed a trend to a heavier liver compared to those from non-supplemented dams. A nutrition by pregnancy rank interaction was not evident (Table 1). Fetal brain/liver ratio was greater in twins compared to singletons (*p* < 0.001) and in fetuses from unsupplemented compared to supplemented ewes (*p* = 0.017), and a trend corresponding to the interaction between factors was observed (Table 1; *p* = 0.052). Singletons had greater GI and tended to have greater BMI compared to twins (Table 1), while maternal plane of nutrition and the interaction between pregnancy rank and maternal plane of nutrition were not significant.

**Figure 2:**
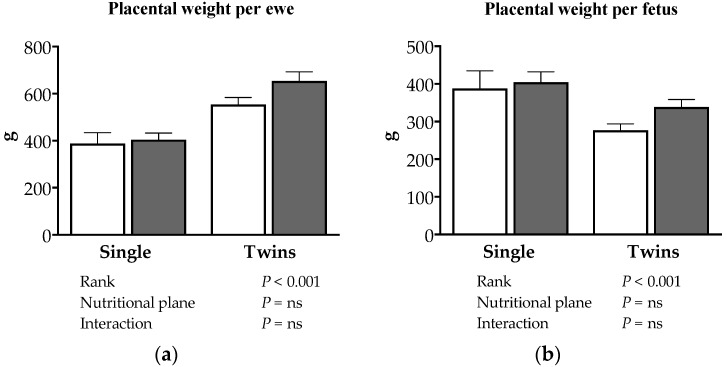
(**a**) Placental weight per ewe. (**b**) Plcental weight per fetus. Placental outcome at 140 days of pregnancy in ewes bearing single and twin fetuses, maintained under adequate or deprived nutritional planes. The empty bars correspond to unsupplemented animals. The gray bars correspond to animals fed with natural pasture plus concentrate supplementation. The corresponding effects and their statistical significance are presented as footnote in each figure.

Total (per ewe) and individual (per fetus) placental weights were affected by pregnancy rank (*p* < 0.001), with greater total placental weight per ewe in twin than in single pregnancies (Figure 2a), whilst the individual placental weight was lower for twins compared to singletons (Figure 2b). No effect of nutritional plane nor interaction between pregnancy rank and nutritional plane were observed. A significant correlation between fetal weight and the weight of its individual placenta was observed (r = 0.632, *p* < 0.001), whereby smaller fetuses had smaller placentae.

### 3.3. Effects of Nutrition and Twinning on Maternal and Feto-Placental Oxygenation Status

The data for maternal and feto-placental oxygenation status are shown in Table 2. Maternal arterial blood gases showed subtle differences among groups, and no significant effects of pregnancy rank, maternal nutritional plane, or their interaction were observed. As regards feto-placental blood (umbilical vein cord), maternal nutritional plane and pregnancy rank (*p* < 0.01 in both cases) affected fetal PO_2_, and single and supplemented fetuses showed higher PO_2_ than their twin and non-supplemented counterparts. No interaction between factors was observed. The SatHb showed analogous effects to those found when considering PO_2_. Hemoglobin concentration was increased in twins compared to singletons (*p* < 0.05), but no significant effect of maternal nutritional plane nor interaction between maternal nutritional plane and pregnancy rank were observed. Consistent with the above, the calculated O_2_ content was affected by maternal nutritional plane and pregnancy rank (*p* < 0.05 in both cases) in a similar manner to PO_2_ and SatHb. Cord blood PCO_2_ and pH were not affected by maternal nutritional plane or pregnancy rank, and no interaction between these factors was observed (Table 2).

Cord blood PO_2_ and O_2_ content were positively correlated with fetal body weight (r = 0.459, *p* < 0.01 and r = 0.446, *p* < 0.01, respectively), crown–rump length (r = 0.321, *p* < 0.01 and r = 0.237, *p* < 0.05), thorax perimeter (r = 0.433, *p* < 0.005 and r = 0.489, *p* < 0.001, respectively), forelimb length (r = 0.548, *p* < 0.001 and r = 0.544, *p* < 0.001, respectively), hind limb length (0.433, *p* < 0.005 and r = 0.384, *p* < 0.01, respectively), and placental weight per fetus (r = 0.481, *p* < 0.001 and r = 0.381, *p* < 0.05).

### 3.4. Effects of Nutrition and Twinning on Feto-Placental Stress Biomarkers

**Figure 3:**
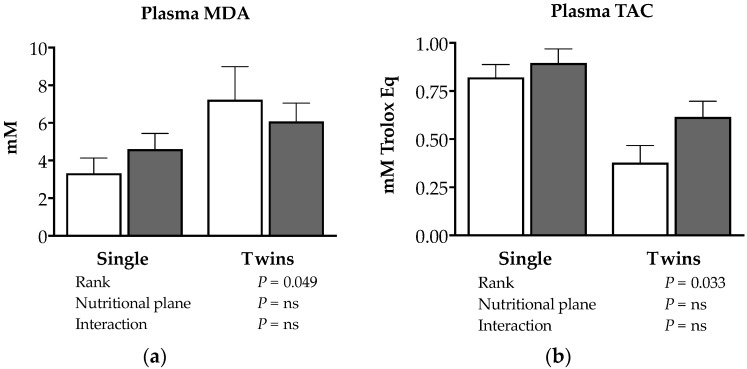
(**a**) Plasma MDA. (**b**) Plasma TAC. Antioxidant status of venous cord blood plasma at 140 days of gestation in ewes bearing single and twin fetuses, maintained under adequate or deprived nutritional planes. The mpty bars correspond to unsupplemented animals. The gray bars correspond to animals fed with natural pasture plus concentrate supplementation. The corresponding effects and their statistical significance are presented as footnote in each figure. MDA: malondialdehyde; TAC: total antioxidant capacity.

Indexes of oxidative status in cord blood are shown in Figure 3. Twin fetuses had greater plasma malondialdehyde (MDA) concentrations and decreased total antioxidant capacity (TAC) compared to singletons (*p* < 0.05; Figure 3a,b, respectively). Conversely, plasma MDA and TAC were unaffected by maternal nutritional planes (*p* > 0.05). No significant correlations among oxidative stress biomarkers and fetal or placental traits were observed.

## 4. Discussion

The results of the present study indicate that, in sheep, maternal undernutrition, imposed by commercial grazing conditions used in Patagonia, negatively influences the live weight gain and the body condition score of dams in late gestation and, afterwards, the growth and oxygenation status of their fetuses. Such effects may be corrected with supplementation to meet the maternal nutritional requirements. On the other hand, twinning, compared to singleton pregnancies, negatively affects placental size, fetal growth, oxygenation status, and antioxidant capacity of the fetal-placental unit, increasing, therefore, lipid peroxidation, even when ewes are fed a plane of nutrition to meet their nutritional requirements. These results highlight the importance of hypoxia and oxidative stress as factors involved in fetal growth restriction resulting from twinning and maternal undernutrition and identify them as potential targets for intervention strategies to improve animal health and welfare, as well as their reproductive and productive potential, especially in challenging environments.

In all well-nourished mammals, the maternal increase in live weight occurring during the first two-thirds of pregnancy is mainly caused by fat deposition, associated with both hyperphagia and increased lipogenesis [29]. The increase in live weight occurring during the last third of pregnancy is generally related to the increase in the weight of the conceptus, while there is an accelerated breakdown of the previously accumulated fat deposits to support an adequate fetal development [29]. In the non-supplemented groups of the current trial (ewes consuming only natural pasture), the live weight remained unchanged, and the BCS declined throughout gestation. In the last third of gestation for this group, there was no increase in ewes’ live weight, in spite of the increase in conceptus mass, suggesting substantial mobilization of maternal body reserves. These results indicate that Patagonian prairies (natural pastures) are not able to meet pregnancy’s nutrient requirements. In contrast, sheep supplemented with a concentrate, showed a sustained live weight increase with advancing pregnancy, consistent with a well-nourished pregnancy leading to normal fetal outcomes [30]. Furthermore, the BCS of the supplemented sheep increased until mid-gestation, reaching values between 2.5 and 3.0 which were maintained during the rest of pregnancy in singleton-bearing ewes with only a small decline in twin-bearing ewes. This level of body condition in late pregnancy is considered adequate for optimum lamb birth weight [31]. It is acknowledged that feed consumption during pregnancy was not measured, because of the inability to measure pasture intake in extensive grazing conditions. However, the purpose of the study was to evaluate maternal and fetal parameters under natural commercial grazing conditions in the Chilean Patagonia. Taking into consideration all the aforementioned findings and potential limitations, the results of this study would confirm that non-supplemented sheep under these extensive grazing management conditions in Patagonian rangeland are undernourished.

The effects of twinning on fetal growth have also been studied in sheep. Independently of other concurrent factors, twinning is widely recognized as a detrimental factor for birth weight, with twins showing 13–20% lower weight than singletons [9,10,32,33], although such differences not always reach statistical significance [34]. In agreement with these reports, placental and fetal development were affected in twin pregnancies in the current study, which was associated with a decrease in fetal body weight of around 16% in twins when compared to singletons and an 8% reduced GI, which has been previously shown to be affected in fetuses under restricted conditions [25]. In addition to fetal weight restriction, twinning affects fetal organ development. In our study, liver weight was reduced in twins compared to singletons. The difference was ameliorated by maternal supplementation. In contrast, brain weight was not affected by pregnancy rank or the maternal plane of nutrition, which is in agreement with previous reports [25,35], suggesting that fetal brain growth is less affected under restricted conditions than other organs. Our study confirms the benefits of maternal supplementation on twin fetal growth, considering that the potential outcomes of reduced lambing weight in twins are higher mortality rates [16] and reduced growth [36], which may have economic effects. Therefore, dietary supplementation of pregnant ewes in the Patagonian harsh environment offers a potential intervention strategy, which could result in increased lambing survival rates, due to increased lamb weight. However, further work is required to determine practical maternal dietary supplementation strategies in extensive rangeland conditions and the cost/benefit ratio of such interventions to establish whether such strategies may be cost-effective and feasible.

The effects on placental and fetal development associated with maternal undernutrition and twinning found in the present study were concomitant with changes in the oxygenation and oxidative status of the fetus. To the best of our knowledge, this is the first direct mechanistic study on the relationships among maternal nutrition, twinning, oxygenation, antioxidant/oxidative status, and fetal growth restriction. Both undernutrition and twinning affected oxygenation. However, the antioxidant/oxidative status of the feto-placental units was affected only by twinning. Placental development is known to be affected by maternal undernutrition [37]; nevertheless, twinning has larger effects due to the limitation of uterine space necessary for adequate placental development [33,38]. Abnormal placental development is known to be related to placental insufficiency [39,40], which leads to inadequate transfer of nutrients and oxygen to the fetus and, therefore, to restricted fetal development. Moreover, twins have to share maternal nutrients and oxygen [38], which exacerbates this scenario. Although hypoxia has been previously demonstrated only in triplet sheep pregnancies [21], our results in twins show a significant decrease of the mother’s oxygen supply to the fetuses, with greater oxygen deprivation in twin pregnancies in undernourishment conditions. The levels of PO_2_ measured in our hypoxic animals are certainly above those defined as hypoxic in other studies [41]. This difference may be due to the fact that we evaluated the PO_2_ in umbilical venous blood, where the oxygen content is higher than in fetal arterial blood, as a result of the circulatory shunts present in the fetal circulation. Furthermore, cord PO_2_ levels reported for normal and hypoxic fetuses in the present study are consistent with those described in cord blood for normoxic and hypoxic term human babies, respectively [42]. These changes in fetal PO_2_ levels were independent of the maternal oxygenation status, because maternal PaO_2_ and SatHb showed no differences among groups, although a lighter decline in both parameters was observed as a consequence of the restrained position for surgery. Our study also demonstrates a clear relationship between fetal oxygen supply and developmental traits, with positive correlations between both PO_2_ and O_2_ cont. and placental traits. The negative effect of hypoxia on sheep pregnancies has been extensively studied using animals maintained at high altitude (i.e., at hypobaric hypoxia) [43,44,45] or at low altitude but subject to artificial hypoxia by means of induced placental insufficiency by carunculectomy, embolization, single umbilical artery ligation, or hyperthermia [46], although hyperthermia also diminishes the food intake. One of the main consequences is compromised oxygenation of the fetuses with increased incidence of FGR and lower birth weight. The similar findings at sea-level altitude of the current study highlight the role of hypoxia in inadequate fetal development resulting from maternal nutritional restriction and/or twinning.

Hypoxia may also be related to increased oxidative stress, due to incomplete oxygen reduction, which may negatively affect embryo and fetal development [22,47]. In the present study, twin pregnancies, i.e., those with more compromised placental development, showed lower total antioxidant capacity in cord blood, confirming previous observational studies in sheep [48] and humans [49,50]. One of the main consequences of deficiencies in the antioxidant capacity during pregnancy is an increased lipid peroxidation, as found in our current study (increased cord plasma malondialdehyde). Increased lipid peroxidation, in turns, decreases lipid availability at the feto-placental unit. This is particularly evident for polyunsaturated fatty acids [51,52,53,54], for which impaired placental transfer causes or exacerbates FGR [53,55,56].

In conclusion, the results obtained in the current study demonstrate that both maternal undernutrition and twinning are associated with decreased oxygen supply to the fetuses, which may explain, at least in part, the decreased intrauterine growth. This confirms the transcendental role of maternal nutrition on lamb’s intrauterine growth, which is particularly critical for twin pregnancies. In addition, twinning was associated with a state of oxidative stress of the feto-placental unit, which might also contribute to FGR. These results highlight hypoxia and oxidative stress in FGR fetuses resulting from maternal undernutrition and/or twinning as potential targets to reduce or ameliorate FGR in these situations, using new nutritional (e.g., rumen-protected amino acids or fatty acids; [33,57,58]) or antioxidant (e.g., vitamins C and E; [22,45]) intervention strategies in sheep flocks. Future research is needed to define appropriate strategic supplementation options that are suitable for extensive ovine systems such as the Chilean Patagonia system.

## Figures and Tables

**Table 1:** Fetal outcomes at 140 days of pregnancy in ewes bearing single and twin fetuses, maintained under adequate or deprived nutritional plane.

						*p*-Value	
	SP	SP+S	TP	TP+S	R	NP	R × NP
Fetal morphometry							
Body weight (kg)	4.3 ± 0.2	4.5 ± 0.2	3.4 ± 0.1	4.0 ± 0.1	<0.001	0.025	ns
Thorax perimeter (cm)	34.5 ± 0.4	36.1 ± 0.7	32.3 ± 0.6	34.0 ± 0.5	0.001	0.016	ns
CRL (cm)	44.8 ± 0.7	45.8 ± 0.6	40.9 ± 0.5	43.9 ± 0.6	<0.001	0.006	ns
Forelimb length (cm)	31.5 ± 0.5	31.9 ± 0.4	28.9 ± 0.4	30.8 ± 0.4	<0.001	0.020	ns
Hindlimb length (cm)	37.0 ± 0.5	37.3 ± 0.5	33.9 ± 0.5	35.2 ± 0.5	<0.001	ns	ns
Brain weight (g)	52.9 ± 1.1	52.4 ± 1.5	52.8 ± 1.4	52.0 ± 1.3	ns	ns	ns
Liver weight (g)	113.5 ± 6.3	118.9 ± 11.4	74.3 ± 3.6	90.1 ± 3.8	<0.001	0.073	ns
Brain/liver weight	0.47 ± 0.03	0.46 ± 0.03	0.73 ± 0.04	0.59 ± 0.02	<0.001	0.017	0.052
Fetal growth indexes							
GI	14.3 ± 0.6	14.5 ± 0.5	12.8 ± 0.4	13.5 ± 0.4	0.005	ns	ns
BMI	21.3 ± 0.9	21.4 ± 0.7	20.1 ± 0.6	20.4 ± 0.4	0.056	ns	ns

Data are the mean ± SEM. for fetal morphometry and proportionality indices: G index (GI, body weight (BW)/crown–rump length (CRL)^1.5^), body mass index (BMI, BW/CRL^2.0^), SP: singletons offered only natural pasture; TP: twins offered only natural pasture; SP+S: singletons offered natural pasture plus supplementation to meet nutritional requirements; TP+S: twins offered natural pasture plus supplementation; R: rank effect; NP: nutritional plane effect.

**Table 2:** Effect of pregnancy rank and maternal plane of nutrition on maternal arterial and umbilical vein blood gases at 140 days of gestation in sheep.

						*p*-Value	
	SP	SP+S	TP	TP+S	R	NP	R × NP
Maternal blood							
PaO_2_ (mm Hg)	93.5 ± 2.5	93.1 ± 7.2	90.8 ± 7.8	91.4 ± 5.1	ns	ns	ns
PaCO_2_ (mm Hg)	43.3 ± 2.3	38.9 ± 1.7	44.2 ± 8.2	41.8 ± 2.3	ns	ns	ns
Hb (g/dL)	10.7 ± 0.3	11.0 ± 0.4	10.9 ± 0.3	10.8 ± 0.2	ns	ns	ns
SatHb (%)	91.8 ± 0.3	91.1 ± 1.4	92.3 ± 0.4	91.8 ± 0.3	ns	ns	ns
pH	7.44 ± 0.02	7.46 ± 0.02	7.45 ± 0.04	7.46 ± 0.02	ns	ns	ns
Umbilical cord blood							
PO_2_ (mm Hg)	27.1 ± 1.8	34.1 ± 2.6	22.2 ± 1.7	26.8 ± 1.7	0.004	0.006	ns
PCO_2_ (mm Hg)	43.2 ± 0.8	39.5 ± 1.5	45.5 ± 1.2	43.2 ± 2.0	ns	ns	ns
Hb (g/dL)	9.6 ± 0.2	10.0 ± 0.5	10.9 ± 0.4	10.6 ± 0.3	0.023	ns	ns
SatHb (%)	43.9 ± 4.3	61.0 ± 6.0	36.4 ± 4.1	43.4 ± 4.2	0.025	0.019	ns
pH	7.44 ± 0.01	7.45 ± 0.02	7.43 ± 0.01	7.43 ± 0.01	ns	ns	ns
O_2_ cont. (mL/dL)	6.0 ± 0.4	8.3 ± 0.8	5.6 ± 0.5	6.5 ± 0.5	0.050	0.016	ns

Data are the mean ± SEM. SP: singletons offered only natural pasture; TP: twins offered only natural pasture; SP+S: singletons offered natural pasture plus supplementation; TP+S: twins offered natural pasture plus supplementation; R: pregnancy rank effect; NP: nutritional plane effect; PO_2_: partial pressure of oxygen; PCO_2_: partial pressure of carbon dioxide; Hb: hemoglobin concentration; SatHb: Hb saturation by oxygen (%), O_2_ cont.: oxygen content.
